# Cerebrovascular Reserve Capacity as a Predictor of Postoperative Delirium: A Pilot Study

**DOI:** 10.3389/fsurg.2021.658849

**Published:** 2021-12-21

**Authors:** Moa Bydén, Anna Segernäs, Hans Thulesius, Farkas Vanky, Eva Ahlgren, Johan Skoog, Helene Zachrisson

**Affiliations:** ^1^Department of Clinical Physiology and Department of Health, Medicine and Caring Sciences, Linköping University, Linköping, Sweden; ^2^Primary Health Care Center in Linköping and Department of Health, Medicine and Caring Sciences, Linköping University, Linköping, Sweden; ^3^Department of Clinical Sciences, Malmö, Lund University Faculty of Medicine, Lund, Sweden; ^4^Department of Medicine and Optometry, Linnaeus University Faculty of Health Social Work and Behavioral Sciences, Kalmar, Sweden; ^5^Department of Thoracic and Vascular Surgery and Department of Health, Medicine and Caring Sciences, Linköping University, Linköping, Sweden

**Keywords:** delirium, cerebrovascular reactivity, transcranial Doppler, cardiopulmonary bypass, mini mental state examination, A Quick Test of cognitive speed

## Abstract

**Introduction:** Postoperative delirium is a common complication after cardiac surgery with cardiopulmonary bypass (CPB). Compromised regulation of the cerebral circulation may be a predisposing factor for delirium. However, the potential relationship between cerebrovascular reserve capacity and delirium is unknown. The aim of this study was to investigate if impaired cerebrovascular reserve capacity was associated with postoperative delirium.

**Methods:** Forty-two patients scheduled for cardiac surgery with CPB were recruited consecutively. All patients underwent preoperative transcranial Doppler (TCD) ultrasound with calculation of breath-hold index (BHI). BHI < 0.69 indicated impaired cerebrovascular reserve capacity. In addition, patients were examined with preoperative neuropsychological tests such as MMSE (Mini Mental State Examination) and AQT (A Quick Test of cognitive speed). Postoperative delirium was assessed using Nursing Delirium Screening Scale (Nu-DESC) in which a score of ≥2 was considered as delirium.

**Results:** Six patients (14%) scored high for postoperative delirium and all demonstrated impaired preoperative cerebrovascular reserve capacity. Median (25th−75th percentile) BHI in patients with postoperative delirium was significantly lower compared to the non-delirium group [0.26 (−0.08–0.44) vs. 0.83 (0.57–1.08), *p* = 0.002]. Preoperative MMSE score was lower in patients who developed postoperative delirium (median, 25th−75th percentile; 26.5, 24–28 vs. 28.5, 27–29, *p* = 0.024). Similarly, patients with postoperative delirium also displayed a slower performance during the preoperative cognitive speed test AQT color and form (mean ± SD; 85.8 s ± 19.3 vs. 69.6 s ± 15.8, *p* = 0.043).

**Conclusion:** The present findings suggest that an extended preoperative ultrasound protocol with TCD evaluation of cerebrovascular reserve capacity and neuropsychological tests may be valuable in identifying patients with increased risk of developing delirium after cardiac surgery.

## Introduction

Postoperative delirium is common after cardiac surgery with a prevalence of 11–52% depending on patient selection and evaluation method (1). The complication is associatedwith increased length of hospital stay and worse patients' outcomes (2). Predisposing factors includes, for example, advanced age, diabetes mellitus, atrial fibrillation, cognitive impairment, duration of surgery (1), but the pathophysiology is not fully understood. During cardiopulmonary bypass (CPB) surgery, reduced cerebral perfusion may play an important role in the pathogenesis (3). Adequate cerebral blood flow (CBF) depends on sufficient blood supply, and patients with carotid stenosis of > 50% have been suggested to be more prone to develop postoperative delirium (4). However, CBF is also protected by an autoregulatory mechanism designed to provide a stable blood flow despite changes in the perfusion pressure (5, 6), and there are indications that cerebral autoregulation may be impaired both pre- and postoperatively in patients developing delirium after CPB (7, 8). Another important mechanism involved in the regulation of CBF is cerebrovascular reactivity, which represents the ability of cerebral vessels to locally constrict or dilate in response to vasoactive stimuli such as pO_2_ and pCO_2_ (9). For example, an increase in pCO_2_ normally leads to vasodilation, but when the cerebrovascular reserve capacity is reduced, the flow becomes more dependent on cerebral perfusion pressure and autoregulation. This means that regional impairments in cerebrovascular reserve capacity can lead to regional hypo- or hyper perfusion even in settings when cerebral autoregulation and global CBF are normal (9). Cerebrovascular reserve capacity can easily and non-invasively be evaluated using transcranial Doppler (TCD) ultrasound and the parameter is of interest since fluctuations in arterial CO_2_ tension are associated with major surgery (10). Accordingly, reduced cerebrovascular reserve capacity in patients with or without carotid stenosis could theoretically be related to a higher risk of hypoperfusion during CPB surgery, and thus postoperative delirium. The aim of this study was to investigate if preoperative ultrasound examinations, including evaluation of carotid stenosis and cerebrovascular reserve capacity, were associated with development of delirium after cardiac surgery with CPB. An additional aim was to evaluate if preoperative cognitive tests, such as the Mini-Mental State Examination (MMSE) and A Quick Test of cognitive speed (AQT), were related to postoperative delirium. We hypothesized that patients with postoperative delirium would have reduced preoperative cerebrovascular reserve capacity and lower cognitive results based on MMSE and AQT.

## Materials and Methods

### Participants

Forty-two patients who underwent cardiac surgery with cardiopulmonary bypass (CPB) at the Department of Cardiothoracic surgery, Linköping University Hospital, Sweden were during a period of 12 months consecutively included in the study. In accordance with our inclusion criteria, all patients needed to be at least 59 years old with no history of dementia or severe psychiatric disease. Other exclusion criteria were emergency procedures, inability to complete the neuropsychological tests (deafness, blindness, language difficulties), and patients with an inadequate temporal bone window for the TCD examination. The patients were scheduled for coronary artery bypass graft (CABG) surgery, aortic valve replacement (AVR) surgery, mitral valve replacement (MVR) surgery or mitral plastic surgery, aortic aneurysm graft surgery or combined surgery (CABG and AVR). Six patients were first included in the study and later excluded; one had language difficulties, one because of scheduling problems and two patients declined further participation after the preoperative tests. One patient underwent a transcatheter aortic valve implantation (TAVI) instead of an AVR and was therefore excluded and one patient had a severe perioperative stroke and was not postoperatively evaluated with Nu-DESC. Medical, health and functional data were collected by interviewing the patients in connection to their neuropsychological tests. Demographic variables and data regarding the operative procedures, risk factors and laboratory values were collected in patients' medical records and in a local database at the cardiothoracic department. The study was approved by the regional ethical review board in Linköping, Sweden, and all participants signed a written informed consent in accordance with the Declaration of Helsinki.

### Delirium Assessment and Neuropsychological Tests

The Nursing Delirium Screening Scale (Nu-DESC) was used to assess delirium (11). The Nu-DESC detects delirium by assessing disorientation, inappropriate behavior, inappropriate communication, illusions/hallucinations, psychomotor retardation, each graded between 0 and 2 (0 = absent, 1 = mild, 2 = severe), with a maximum score of 10. A score of ≥2 was considered as postoperative delirium in this study. Neuropsychological tests, MMSE and AQT, were used to assess the patients' cognitive function. The MMSE assesses orientation in time and place, working memory and delayed memory, attention, language, and visuospatial ability. The maximum score is thirty points (range 0–30), with lower scores denoting more impaired cognition. AQT consists of 40 visual stimuli as geometric figures–circles, squares, rectangles, or triangles, colored red, black, yellow, or blue. In this study, a short-form AQT was used comprised of two parts: form-naming and color and form-naming which measures the patients' perceptual function and cognitive speed. Cut-off scores for abnormal performance for AQT form is > 35 s and AQT color and form > 70 s (12).

### Carotid Duplex Ultrasound

Thirty-nine patients underwent bilateral carotid duplex ultrasonography examinations the day before operation or during postoperative care using ACUSON S2000 TM ultrasound system (Siemens Medical Solutions USA, Inc., California, USA). The European Carotid Surgery Trial (ECST) criteria was used to determine the degree of carotid stenosis based on peak systolic velocity over the stenosis (13). The stenosis were categorized in five grades: <50%, 50–69%, 70–79%, 80–99% and occlusion. The examiner also estimated if the plaque surfaces were ulcerated and plaques were classified as <2 mm. The echogenicity of the plaques was evaluated as well.

### Transcranial Doppler Ultrasound

**Figure 1:**
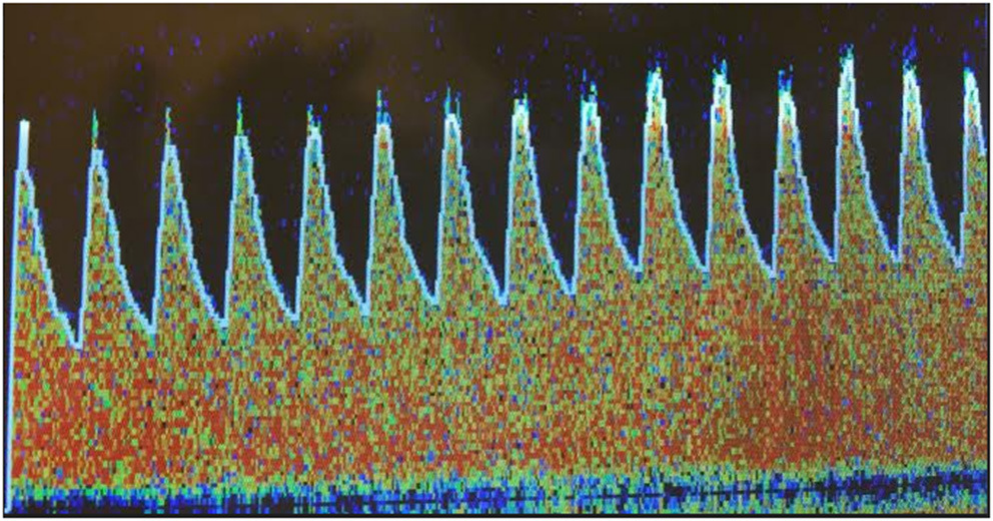
Example of transcranial Doppler ultrasound of middle cerebral artery showing increased mean flow velocity during breath-hold test as a sign of preserved cerebrovascular reserve capacity.

Forty-two patients underwent preoperative TCD of middle cerebral artery (MCA), using a 2-MHz probe (Nicolet SONARA TCD system, Natus Medical Inc, CA, USA). A breath-hold test was performed in order to evaluate the ability of the cerebral vessels to dilate in response to increased pCO_2_, i.e., their cerebrovascular reserve capacity. The test was conducted based on the description of Markus et al. (14). Peak systolic velocity (PSV) and end diastolic velocity (EDV) were recorded at baseline, while the patients were breathing normally, and the mean flow velocity (MFV) was calculated based on the PSV and EDV values. The patients then held their breath for 30 s and the maximum PSV and EDV values following the breath-hold were used to determine MFV. Breath-hold index (BHI) was calculated as the percentage increase in MFV divided by the time for which the subject held their breath (Figure 1) (14). A BHI of ≥0.69 implies preserved cerebrovascular reserve capacity and a BHI of <0.69 indicates impaired cerebrovascular reserve capacity (15). All patients without carotid stenosis were evaluated with TCD of the right MCA. If carotid ultrasound displayed stenosis >50%, bilateral TCD of MCA were conducted and the lowest BHI-value was used.

### Anesthesia and Operative Procedure

After an overnight's fast drugs were withheld with the exception of betablockers and calcium antagonists. Premedication consisted of paracetamol and oxazepam. Anesthesia was induced with thiopentone 1 mg/kg body weight and fentanyl 30 μg/kg body weight. Rocuronium was used for neuromuscular blockade, 50 mg at induction and 30 mg at sternal closure. Anesthesia was maintained with fentanyl and isoflurane. Antibiotics were given as prophylaxis 30 min before skin incision. Heparin was given as recommended by HepCon and activated clotting time was maintained ≥480 s. Phenylephrine was added if mean arterial pressure (MAP) fell < 50 mmHg. The surgical procedures included the following phases: (1) surgery start, skin incision; (2) graft harvesting in CABG procedures; (3) administration of Heparin; (4) establishment of the ECC circuit with insertion of the aortic and venous cannulas when blood pressure (BT) was <100 mmHg, and scan of the ascending aorta after epiaortic ultrasound; (5) start of CPB, time noted in the anesthesia record. Moderate hemodilution (hematocrit 20–25%) and mild hypothermia (33–36 C°) were usually employed; (6) aortic cross clamping and administration of cardioplegic solution, repeatedly, every 20 min for myocardial protection; (7) the cardiac surgery procedure was performed; (8) aortic cross clamp removal; (9) termination of CPB, time noted in the anesthesia record, administration of phenylephrine, adrenaline, noradrenaline when needed; (9) aortic decannulation, when BT < 100 mmHg and administration of protamine. The procedure was performed with non-pulsatile flow during CPB.

### Postoperative Care

The nursing staff in the ICU and the general ward evaluated the patients' degree of confusion and delirium during the postoperative care, three times daily at the end of their work shift, using the Nu-DESC. The staff who made the registrations were well acquainted with the Nu-DESC instrument in clinical routine.

### Statistical Analysis

Statistical analyses were performed using SPSS statistics 27 for Windows (IBM, Armonk, NY, USA). Continuous normally distributed data were compared by unpaired Student's *t*-test and Mann-Whitney U-test was used when data were ordinal or not normally distributed. Categorical variables were analyzed using Fisher's exact test. Spearman's correlation coefficient was used for correlation analyses. Statistical significance was defined at *p* < 0.05.

## Results

### Patient Characteristics

Forty-two patients were assessed for delirium. Six of these (14%), one woman and five men, developed postoperative delirium based on Nu-DESC with a total score of ≥ 2. None of the patients were delirious prior to the surgery according to Nu-DESC. No differences in age, cardiovascular comorbidity, duration of surgery or time with extracorporeal circulation were detected between patients with postoperative delirium compared to the non-delirium group (Table 1). Four patients (9%) were re-operated and two of them developed postoperative delirium.

**Table 1:** Pre- and intraoperative characteristics of patients with and without postoperative delirium.

	**No delirium** **(*n* = 36)**	**Delirium** **(*n* = 6)**	***p*** **-value**
Age, mean ± SD	70.4 ± 5.8	69.7 ± 6.4	0.77
Females, *n* (%)	4 (11.1%)	1 (16.7%)	0.56
Type of surgery CABG, *n* (%)	18 (50%)	5 (83.3%)	0.2
Valve replacement, *n* (%)	12 (33.3%)	1 (16.7%)	0.65
Combined, *n* (%)	6 (16.7%)		0.57
Atrial fibrillation, *n* (%)	2 (5.6%)	1 (16.7%)	0.38
Hypertension, *n* (%)	22 (61.1%)	5 (83.3%)	0.40
Diabetes, *n* (%)	7 (19.4%)	2 (33.3%)	0.59
Cerebrovascular disease, *n* (%)	2 (5.6%)		1
Peripheral vascular disease, *n* (%)	2 (5.6%)	2 (33.3%)	0.091
Heart failure NYHA I, *n* (%)	2 (5.6%)	1 (16.7%)	0.38
NYHA II, *n* (%)	20 (55.6%)	3 (50%)	1
NYHA III, *n* (%)	12 (33.3%)	2 (33.3%)	1
NYHA IV, *n* (%)	2 (5.6%)		1
Duration of surgery (min), mean ± SD	178 ± 46	191.5 ± 27.7	0.49
Extracorporeal circulation (min), mean ± SD	91.91 ± 31.3	87.8 ± 18.7	0.76

*CABG, coronary bypass graft; NYHA, New York Heart Association class*.

### Cerebrovascular Reserve Capacity

**Figure 2:**
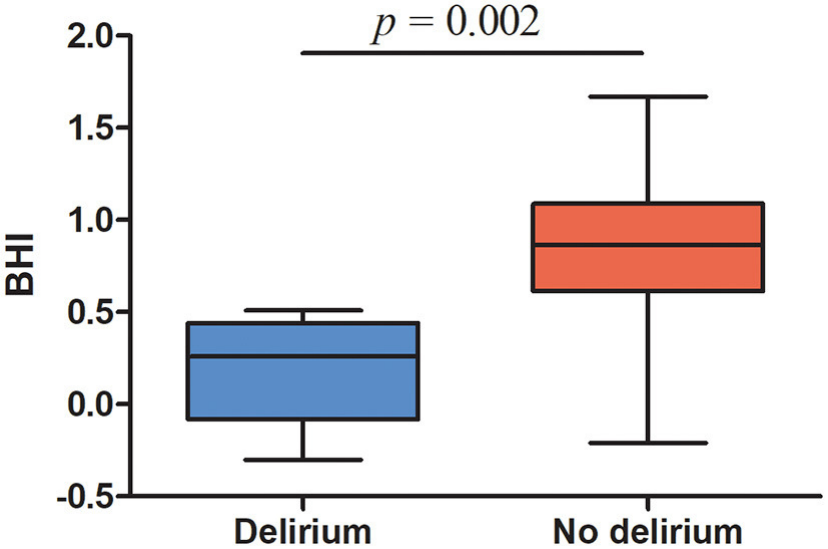
Preoperative breath-hold index in patients with postoperative delirium and patients without delirium. Top and bottom ends of each box, 25th and 75th percentiles; horizontal line in the middle of the boxes, median; whiskers, lowest and highest values.

Patients were able to hold their breath for 30 s during the breath-hold test. All of the six patients diagnosed with postoperative delirium displayed BHI values below <0.69, indicating impaired cerebrovascular reserve capacity (median, range, 0.26, −0.3 −0.51). Median (25th−75th percentile) BHI in patients with postoperative delirium was significantly lower compared to the non-delirium group 0.26 (−0.08–0.44) vs. 0.83 (0.57–1.08), *p* = 0.002, Figure 2, Table 2). In total, cerebrovascular reserve capacity was impaired in 18 of the 42 patients (43%). BHI <0.69 had a 100% sensitivity and 64% specificity in predicting postoperative delirium with a positive predictive value of 32% and negative predictive value of 100%. BHI was positively correlated to MMSE (rho = 0.396, *p* = 0.011), but no correlations were found with AQT color and form (rho = −0.203, *p* = 0.21) or AQT form (rho = −0.082, *p* = 0.62).

**Table 2:** Preoperative Transcranial Doppler ultrasound, Carotid Duplex ultrasound and neuropsychological tests in patients with and without postoperative delirium.

	**No delirium** **(*n* = 36)**	**Delirium** **(*n* = 6)**	***p*** **-value**
Transcranial Doppler ultrasound BHI, median (IQR)	0.26 (−0.08–0.44)	0.83 (0.57–1.08)	0.002
Carotid Duplex ultrasound ICA >50% stenosis, n (%)	5 (14%)	1 (17%)	1.0
Neuropsychological tests AQT color-form, sec mean (SD)	69.6 ± 15.8	85.8 ± 19.3	0.043
AQT form, mean (SD)	44.5 ± 9.0	38.5 ± 6.7	0.059
MMSE score, median (IQR)	28.5 (27–29)	26.5 (24–28)	0.024

*ICA, internal carotid artery; AQT, A Quick Test of cognitive speed; MMSE, Mini-Mental State Examination; IQR, interquartile range*.

### Carotid Duplex Ultrasonography

Six patients displayed stenosis of >50% in left and/or right ICA. Of these patients, five had a stenosis of 50–69% and one had a stenosis of 70–79%. No differences in respect to developing postoperative delirium were found between patients with ICA stenosis >50% and patients with no stenosis (*p* = 1, Table 2). Similarly, no differences in BHI were detected between patients with ICA stenosis >50% compared to those with no stenosis (median, 25th−75th percentile; 0.71, 0.18–0.98 vs. 0.82, 0.48–1.11, *p* = 0.61).

### MMSE and AQT

Preoperative MMSE scores were significantly lower among patients who developed postoperative delirium (median, 25th−75th percentile; 26.5, 24–28 vs. 28.5, 27–29, *p* = 0.024, Table 2). Patients who developed postoperative delirium also displayed a slower performance during the preoperative cognitive speed test AQT color and form (mean ± SD; 85.8 s ± 19.3 vs. 69.6 s ± 15.8, *p* = 0.043, Table 2), and there was a tendency toward a slower performance during the AQT form (mean ± SD; 44.5 s ± 9.0 vs. 38.5 s ± 6.7, *p* = 0.059, Table 2). However, abnormal AQT color and form times (>70 s) were not associated with postoperative delirium (*p* = 0.34) or carotid stenosis >50% (*p* = 0.40).

## Discussion

The present study demonstrated reduced preoperative cerebrovascular reserve capacity in patients who developed delirium after CPB surgery. Further, patients developing postoperative delirium also had lower scores on preoperative cognitive function tests such as MMSE and AQT color and form.

Reductions in CBF and perfusion have been related to delirium (16). Studies have indicated that both pre- and postoperative impairments of the brains capacity to preserve a stable CBF despite changes in cerebral perfusion pressure, i.e., cerebral autoregulation, may be associated with an increased risk for delirium after surgery (7, 8). Although no distinctive cause-effect relationship has been established, reduced autoregulation might contribute to hypoperfusion when blood pressure is low during CPB, as well as hyperperfusion if blood pressure exceeds the higher limit of autoregulation (17, 18). However, some patients demonstrate intact cerebral autoregulation and still develop postoperative delirium whereas other may display impaired autoregulation but do not develop delirium (8, 19). As such, other factors involved in the regulation of CBF may also be important in the pathogenesis of delirium. One possible, but less studied factor is cerebrovascular reserve capacity.

The frequency of postoperative delirium in the present study was 14%, which is a relatively low number compared to other reports (20). Stringent surgical and nursing care protocols may be a part of the explanation. However, delirium could be underdiagnosed when using Nu-DESC alone, particularly hypoactive delirium (21). Nevertheless, Nu-DESC is a validated easy-to-deploy delirium-screening tool in clinical practice (22), which has been used at our department for several years meaning that the nurses who evaluated the patients were all well acquainted with the instrument. Preoperative evaluation of BHI displayed significantly lower values in patients developing delirium after surgery, and all patients with postoperative delirium demonstrated an abnormal preoperative cerebrovascular reserve capacity (Figure 1). Cerebrovascular reserve capacity represents, on a regional scale, the capacity of cerebral vessels to change vascular tone depending on the vasoactive stimuli (23). The underlying mechanisms between impaired cerebrovascular reserve capacity and postoperative delirium are not clear but alteration in pCO_2_ have profound effects on CBF (24). Changes from hypo- to normocapnia or from normo- to hypercapnia may occur during CBP surgery, and it has been suggested that these alterations can result in intracranial steal in vulnerable individuals (10, 24, 25), i.e., the redistribution of blood flow from regions with exhausted cerebrovascular reserve capacity to areas with preserved vasodilatory capacity (26). In addition to partial pressure of arterial CO_2_ and O_2_, other localized stimuli, such as signaling messages and metabolites released from astrocytes and neurons as well as pH, blood viscosity and temperature affects CBF (9). Perioperative parameters were not assessed in the present study although during the standardized CBP procedure, cerebral oximetry was used to detected changes in CBF and the minute ventilation was corrected to maintain normocapnia in order to keep CBF stable.

In agreement with earlier studies, we found that a lower BHI was associated with lower scores on MMSE (23). Reduced cerebrovascular reserve capacity has been linked to cognitive dysfunction and decreased cognitive function could be a consequence of chronic hypoperfusion due to exhausted cerebrovascular reserve capacity (23, 27). Cognitive impairment is a predisposing factor for postoperative delirium and might not be favorable combined with impaired cerebral hemodynamic (1). In line with these findings, patients who developed delirium demonstrated lower MMSE scores during the preoperative evaluation. Patients with postoperative delirium also presented with a slower performance during the preoperative cognitive speed test AQT color and form. However, no correlation between BHI and AQT color and form was found and abnormal AQT color and form times were not associated with postoperative delirium.

Six patients were diagnosed with carotid stenosis in the present study. Previous studies have associated carotid stenosis with reduced BHI (28–30), as well as increased risk of developing postoperative delirium (4). It has been suggested that decreased cerebral perfusion pressure may lead to limited capacity to increase CBF in response to vasodilatory stimuli because arterioles are already dilated and not able to dilate much further (31, 32). However, we detected no differences in BHI in patients with ICA stenosis > 50% compared to those with no stenosis. Similarly, patients with stenosis did not develop postoperative delirium in a higher frequency compared to those without stenosis. The discrepancy between our findings and previous studies can possibly be explained by the relatively low number of individuals with delirium and/or stenosis in our study. It should be noted that duplex sometimes underestimates the degree of carotid stenosis due to a developed collateral circulation which decreases blood flow velocity over the stenosis (33). As such, cerebrovascular reserve capacity could allow complementary information about cerebral hemodynamic and collateral circulation in addition to information about the degree of carotid stenosis evaluated by duplex (34). In total, cerebrovascular reserve capacity was impaired in 18 of the 42 patients (43%). The reason for decreased BHI in our study is not completely known, but may be explained by the high number of patients with heart failure. Heart failure has been associated with reduced cerebrovascular reactivity (35), and a recent study showed that 57% of the patients with heart failure displayed an BHI less than a clinically considered cut-off value of 0.6 (36). It has been suggested that reductions in cardiac output could be compensated by lowering the resistance in brain arterioles, however, such compensation will minimize any further dilation and thus alter the cerebrovascular reactivity (35). This has been observed even in patients with rather compensated heart failure (NYHA II) (35).

Preoperative detection of patients with a high risk of developing delirium after surgery is important and our findings suggest that impaired cerebrovascular reserve capacity may be a predisposing factor. However, not all patients with abnormal cerebrovascular reserve capacity developed postoperative delirium further highlighting the multifactorial etiology. Thus, further, and larger, studies are needed to evaluate cerebrovascular reserve capacity in combination with other measurements, e.g., preoperative cognitive function tests. It seems reasonable that such a combination may provide more information compared to a single parameter. Thus, although the clinical significance of measuring cerebrovascular reserve capacity preoperatively is not established, our study provides a base for further investigations where cerebrovascular reserve capacity, quantified by BHI, could be one important parameter.

## Limitations

TCD measures CBF velocity as a surrogate measure of CBF based on the assumption that the diameter of MCA does not significantly change during hypercapnic test. However, previous data indicate that TCD can be used as an accurate estimate of blood flow (37). BHI is associated with questions concerning variability and compared to other neuroimaging modalities TCD has a poor spatial resolution for CBF and cerebrovascular reserve capacity. Yet, TCD is readily available, inexpensive, non-invasive, and ideal for repeated measurements if necessary and BHI is simple to calculate, making it an interesting preoperative screening parameter compared to other neuroimaging modalities (38). In connection with the evaluation of BHI, a general examination of MCA, anterior cerebral artery and posterior cerebral artery was performed without signs of high-grade stenosis. However, no complete investigation of the patency of intracranial arteries were conducted. Patients' drug treatment was not registered, meaning that the possible effects of anti-hypertensive medication and beta-blockers on BHI were not evaluated. Patients were assessed using Nu-DESC, which can lead to underestimation of postoperative delirium, particularly hypoactive delirium. The present pilot study was small with relatively few participants developing postoperative delirium. Based on this, no multivariate models could be calculated which is a limitation since development of postoperative delirium is multifactorial in its nature. Thus, the presented results need further validation in larger studies.

## Conclusions

Preoperative cerebrovascular reserve capacity was significantly lower in patients who developed postoperative delirium, and all patients diagnosed with delirium demonstrated impaired cerebrovascular reserve capacity before surgery. Patients with delirium also showed lower MMSE scores and slower AQT color and form reading times during preoperative evaluations. The results of the TCD examinations suggest that cerebrovascular reserve capacity could be valuable in identifying patients at risk of developing postoperative delirium after cardiac surgery with CPB. However, further and larger studies are needed to validate the predictive value of preoperative impaired cerebrovascular reserve capacity and the risk of developing delirium after surgery.

## Data Availability Statement

The raw data supporting the conclusions of this article will be made available by the authors, without undue reservation.

## Ethics Statement

The studies involving human participants were reviewed and approved by the Regional Ethical Review Board in Linköping, Sweden. The patients/participants provided their written informed consent to participate in this study.

## Author Contributions

HZ, AS, HT, and EA contributed to conception and design of the study. HZ was main responsible for the project. MB, AS, HT, FV, EA, JS, and HZ analyzed and/or interpreted the data. MB wrote the first draft of the manuscript. All authors contributed to manuscript revision, read, and approved the submitted version.

## Funding

This research was funded by ALF Grants, Region Ostergotland [LIO-700491].

## Conflict of Interest

The authors declare that the research was conducted in the absence of any commercial or financial relationships that could be construed as a potential conflict of interest.

## Publisher's Note

All claims expressed in this article are solely those of the authors and do not necessarily represent those of their affiliated organizations, or those of the publisher, the editors and the reviewers. Any product that may be evaluated in this article, or claim that may be made by its manufacturer, is not guaranteed or endorsed by the publisher.
